# Supplementary material for: A new model for predicting the outcome and effectiveness of drug therapy in patients with severe fever with thrombocytopenia syndrome: A multicenter Chinese study
Source: PLoS Negl Trop Dis. 2023 Mar 6;17(3):e0011158. doi: 10.1371/journal.pntd.0011158 (PMC10019728; doi:10.1371/journal.pntd.0011158)
Supplement: S1 Table — (DOCX) [file pntd.0011158.s001.docx]

**Appendices**

**Table S1 Comparison of clinical features among patients in the modeling and validation groups (% or range)**

| **Characteristics** | Modeling group (n=161) | Validation group  (n=216) | *P* Value |
| --- | --- | --- | --- |
| **Demographic feature** |  |  |  |
| Male | 63 (39.13) | 91 (42.12) | 0.558 |
| Age, year | 64 (54, 69) | 63 (54, 70) | 0.830 |
| **Clinical manifestation on admission** | | | |
| Headache | 74 (45.96) | 36 (16.67) | <0.001 |
| Fatigue | 40 (24.84) | 22 (10.19) | <0.001 |
| Myalgia | 55 (34.16) | 31 (14.35) | <0.001 |
| Nausea and Vomiting | 43 (26.71) | 104 (48.15) | <0.001 |
| Lymphadenopathy | 95 (59.01) | 158 (73.15) | 0.004 |
| Gastrointestinal bleeding | 102 (63.35) | 172 (79.63) | <0.001 |
| Ecchymosis | 93 (57.76） | 167 (77.31) | <0.001 |
| Neurologic symptoms | 33 (20.50） | 43 (19.9） | 0.888 |
| Pancreatitis | 54 (33.54） | 108 (100） | 0.001 |
| Accompanied infection | 84 (52.17) | 141 (65.28) | 0.010 |
| **Laboratory tests** |  |  |  |
| SFTS IgM (positive) | 103 (63.98) | 105 (48.61) | 0.013 |
| SFTS IgG (positive) | 21 (13.04) | 25 (11.57) | 0.794 |
| SFTSV RNA  (lg, copies/ml） | 3.89 (3.02, 5.02) | 4.00 (3.30, 5.20) | 0.193 |
| White blood cells（×10^9^/L） | 2.07 (1.43, 3.58) | 2.43 (1.43, 4.14) | 0.272 |
| Neutrophils（×10^9^/L） | 1.17 (0.77, 2.11) | 1.29 (0.78, 2.41) | 0.303 |
| Lymphocytes（×10^9^/L） | 0.54 (0.40, 1.11) | 0.64 (0.40, 1.16) | 0.512 |
| Eosinophil (%) | 0.00 (0.00, 0.10) | 0.00 (0.00, 0.10) | 0.007 |
| Red blood cells（×10^9^/L） | 4.20 (3.93, 4.56) | 4.22 (3.92, 4.57) | 0.905 |
| Hemoglobin (g/L) | 127 (117, 138) | 128(116, 137) | 0.926 |
| Platelets（×10^9^/L） | 48 (31, 65) | 45 (32, 63) | 0.626 |
| ALT(U/L) | 77 (48, 124) | 78 (48, 136) | 0.813 |
| AST(U/L) | 170 (110, 406) | 196.5 (111, 355) | 0.645 |
| Total bilirubin (μmol/L) | 9.6 (7.2, 13.2) | 8.9 (6.7, 12.6) | 0.266 |
| eGFR (ml. min/1.73 m^2^) | 87.97 (68.41, 111.95) | 93.22 (72.28, 114.59) | 0.144 |
| Glucose (mmol/L) | 6.24 (5.38, 7.80) | 6.70 (5.65, 8.39) | 0.034 |
| Calcium(mmol/L) | 1.91 ± 0.15 | 1.94 ± 0.15 | 0.091 |
| CK(U/L) | 413 (234, 835) | 539 (251, 1076) | 0.077 |
| LDH(U/L) | 595 (388, 871) | 917 (538, 2013) | <0.001 |
| PT-INR | 1.03 (0.98, 1.12) | 1.01 (0.95, 1.13) | 0.987 |
| D-D dimer（ng/L） | 3.23 (1.49, 6.70) | 2.84 (1.35, 5.02) | 0.193 |
| Amylase (U/L) | 123 (75, 190) | 132 (89, 205) | 0.169 |
| Lipase (U/L) | 170 (87, 410) | 432 (142, 850) | <0.001 |
| Urinary occult blood (positive) | 49 (30.43) | 15 (6.94) | <0.001 |
| Urine protein (positive) | 91 (56.52) | 10 (4.63) | <0.001 |

Note

Ranges of 2 and above were defined as positive, while ranges of 2 and below were defined as negative for urinary occult blood and urine protein.

OR: Odds ratio; SFTS: Severe fever with thrombocytopenia syndrome; IgM: Immunoglobulin M; IgG: Immunoglobulin G; SFTSV: SFTS virus; ALT: Alanine aminotransferase; AST: Aspartate transaminase; TBIL: Total bilirubin; eGFR: Glomerular filtration rate; CK: Creatinine kinase; LDH: Lactate dehydrogenase; PT-INR: Prothrombin time-internationalization ratio
