# Supplementary material for: A new model for predicting the outcome and effectiveness of drug therapy in patients with severe fever with thrombocytopenia syndrome: A multicenter Chinese study
Source: PLoS Negl Trop Dis. 2023 Mar 6;17(3):e0011158. doi: 10.1371/journal.pntd.0011158 (PMC10019728; doi:10.1371/journal.pntd.0011158)
Supplement: S2 Table — (DOCX) [file pntd.0011158.s002.docx]

**Table S2 Multivariate regression analysis of independent risk factors affecting the prognosis of patients with SFTS in the modeling group**

| Variables | β value | SE | Wald | OR value（95% CI） | *P* value |
| --- | --- | --- | --- | --- | --- |
| Age | 0.070 | 0.028 | 6.210 | 1.073（1.015, 1.134） | 0.013 |
| SFTSV RNA | 0.621 | 0.205 | 9.142 | 1.861 (1.244, 2.784) | 0.002 |
| Gastrointestinal bleeding | 2.048 | 0.540 | 14.372 | 7.755 (2.690, 22.362) | <0.001 |

Note

OR: Odds ratio; CI: Confidence interval; SFTS: Severe fever with thrombocytopenia syndrome; SFTSV: SFTS virus
