# Supplementary material for: A new model for predicting the outcome and effectiveness of drug therapy in patients with severe fever with thrombocytopenia syndrome: A multicenter Chinese study
Source: PLoS Negl Trop Dis. 2023 Mar 6;17(3):e0011158. doi: 10.1371/journal.pntd.0011158 (PMC10019728; doi:10.1371/journal.pntd.0011158)
Supplement: S3 Table — (DOCX) [file pntd.0011158.s003.docx]

**Table S3 Comparison of clinical features among patients in** **the survival and death subgroups of the verification group (% or range)**

| **Characteristics** | Survival group (n=174) | Death group  (n=42) | OR value | *P* value |
| --- | --- | --- | --- | --- |
|  |  |  |  |  |
| **Demographic feature** |  |  |  |  |
| Male | 73 (41.95） | 18 (42.86） | 1.038 | 0.917 |
| Age, year | 61.5 (52, 68) | 70 (64.75, 73.25) | 1.115 | <0.001 |
| **Clinical manifestation on admission** | | | | |
| Headache | 146 (83.91) | 34（80.95） | 0.815 | 0.645 |
| Fatigue | 158 (90.80) | 36 (85.71） | 0.608 | 0.328 |
| Myalgia | 149 (85.63） | 36 (85.71） | 1.007 | 0.989 |
| Nausea and Vomiting | 81 (46.55） | 31 (73.81） | 3.236 | 0.002 |
| Lymphadenopathy | 44 (25.29） | 14 (33.33） | 1.477 | 0.291 |
| Pancreatitis | 81 (45.98） | 27 (64.29） | 2.067 | 0.039 |
| Gastrointestinal bleeding | 20 (11.49） | 24 (57.14） | 10.267 | <0.001 |
| Ecchymosis | 30 (17.24） | 19 (45.24） | 3.965 | <0.001 |
| Secondary infection | 57 (32.76） | 18 (42.86） | 1.539 | 0.217 |
| Neurologic symptoms | 11 (6.32） | 32 (76.19） | 47.418 | <0.001 |
| **Laboratory tests** |  |  |  |  |
| SFTS IgM(positive) | 92（52.87） | 13（30.95） | 0.351 | 0.004 |
| SFTS IgG(positive) | 25 (14.37) | 0（0） | 0.000 | 0.007 |
| SFTSV RNA  (lg, copies/ml） | 3.95 (3.00, 4.57) | 5.98 (5.02, 6.98) | 3.256 | <0.001 |
| White blood cells (×10^9^/L) | 2.51 (1.5, 4.35) | 1.91 (1.29, 3.1) | 0.930 | 0.039 |
| Neutrophils (×10^9^/L) | 1.32 (0.78, 2.59) | 1.18 (0.80, 1.83) | 0.909 | 0.338 |
| Lymphocytes (×10^9^/L) | 0.68 (0.42, 1.21) | 0.51 (0.35, 0.84) | 1.027 | 0.038 |
| Eosinophil (%) | 0.00 (0.00, 0.10) | 0.00 (0.00, 0.04) | 0.945 | 0.616 |
| Red blood cells (×10^9^/L) | 4.26 (3.95, 4.58) | 4.19 (3.77, 4.57) | 0.743 | 0.562 |
| Hemoglobin (g/L) | 128 (116, 138) | 130 (116, 136) | 0.992 | 0.802 |
| Platelets (×10^9^/L) | 49 (35, 65) | 40 (28, 50) | 0.983 | 0.023 |
| ALT(U/L) | 70 (43, 113) | 132 (84, 220) | 1.008 | <0.001 |
| AST (U/L) | 168 (92, 263) | 445 (343, 750) | 1.004 | <0.001 |
| TBIL(μmol/L) | 8.60 (6.60, 12.20) | 10.35 (7.83, 14.10) | 1.017 | 0.022 |
| eGFR (ml.min/1.73 ml^2^) | 94.50 (75.65,120.11) | 87.86 (51.14, 104.31) | 0.987 | 0.012 |
| Glucose (mmol/L) | 6.56 (5.61, 8.16) | 7.60 (5.95, 10.45) | 1.151 | 0.019 |
| Calcium (mmol/L) | 1.96 ± 0.14 | 1.85 ± 0.14 | 0.006 | 0.008 |
| CK(U/L) | 522 (223, 915) | 992 (382, 1886) | 1.000 | 0.001 |
| LDH(U/L) | 801 (476, 1409) | 2094 (996, 3633) | 1.001 | <0.001 |
| PT-INR | 1.02 (0.94, 1.12) | 1.11 (1.03, 1.26) | 56.639 | 0.001 |
| D-D dimer（ng/L） | 2.30 (1.10, 3.94) | 5.74 (3.89, 8.73) | 1.050 | <0.001 |
| Amylase (U/L) | 117 (86, 204) | 159 (114, 213) | 1.000 | 0.075 |
| Lipase (U/L) | 386 (126, 839) | 606 (211, 1034) | 1.000 | 0.118 |
| Urinary occult blood (positive) | 8 (4.60) | 7 (16.67) | 4.150 | 0.006 |
| Urine protein (positive) | 6 (3.45） | 4 (9.52） | 2.947 | 0.093 |

Note

Range of 2 and above were defined as positive, while ranges of 2 and below were defined as negative for urinary occult blood and urine protein.

OR: Odds ratio; SFTS: Severe fever with thrombocytopenia syndrome; IgM: Immunoglobulin M; IgG: Immunoglobulin G; SFTSV: SFTS virus; ALT: Alanine aminotransferase; AST: Aspartate transaminase; TBIL: Total bilirubin; eGFR: Glomerular filtration rate; CK: Creatinine kinase; LDH: Lactate dehydrogenase; PT-INR: Prothrombin time-internationalization ratio
