# Supplementary material for: A new model for predicting the outcome and effectiveness of drug therapy in patients with severe fever with thrombocytopenia syndrome: A multicenter Chinese study
Source: PLoS Negl Trop Dis. 2023 Mar 6;17(3):e0011158. doi: 10.1371/journal.pntd.0011158 (PMC10019728; doi:10.1371/journal.pntd.0011158)
Supplement: S4 Table — (DOCX) [file pntd.0011158.s004.docx]

|  | Modeling group (n=161) | |  | Validation group (n=216) | |
| --- | --- | --- | --- | --- | --- |
|  | Survival group | Death group |  | Survival group | Death group |
| M > 10 | 12 | 4 |  | 10 | 16 |
| M < 10 | 123 | 22 |  | 164 | 26 |
| Sensitivity | 25.0% | |  | 61.5% | |
| Specificity | 84.8% | |  | 86.3% | |

**Table S4 Sensitivity and specificity of the model established by Wang based on our data (Wang et al)**

Note

M= 0.002× AST+ 0.121× Age+ 0.013× Scr

SCr: Serum creatinine
