# Supplementary figures and images for: A new model for predicting the outcome and effectiveness of drug therapy in patients with severe fever with thrombocytopenia syndrome: A multicenter Chinese study
Source: PLoS Negl Trop Dis. 2023 Mar 6;17(3):e0011158. doi: 10.1371/journal.pntd.0011158 (PMC10019728; doi:10.1371/journal.pntd.0011158)

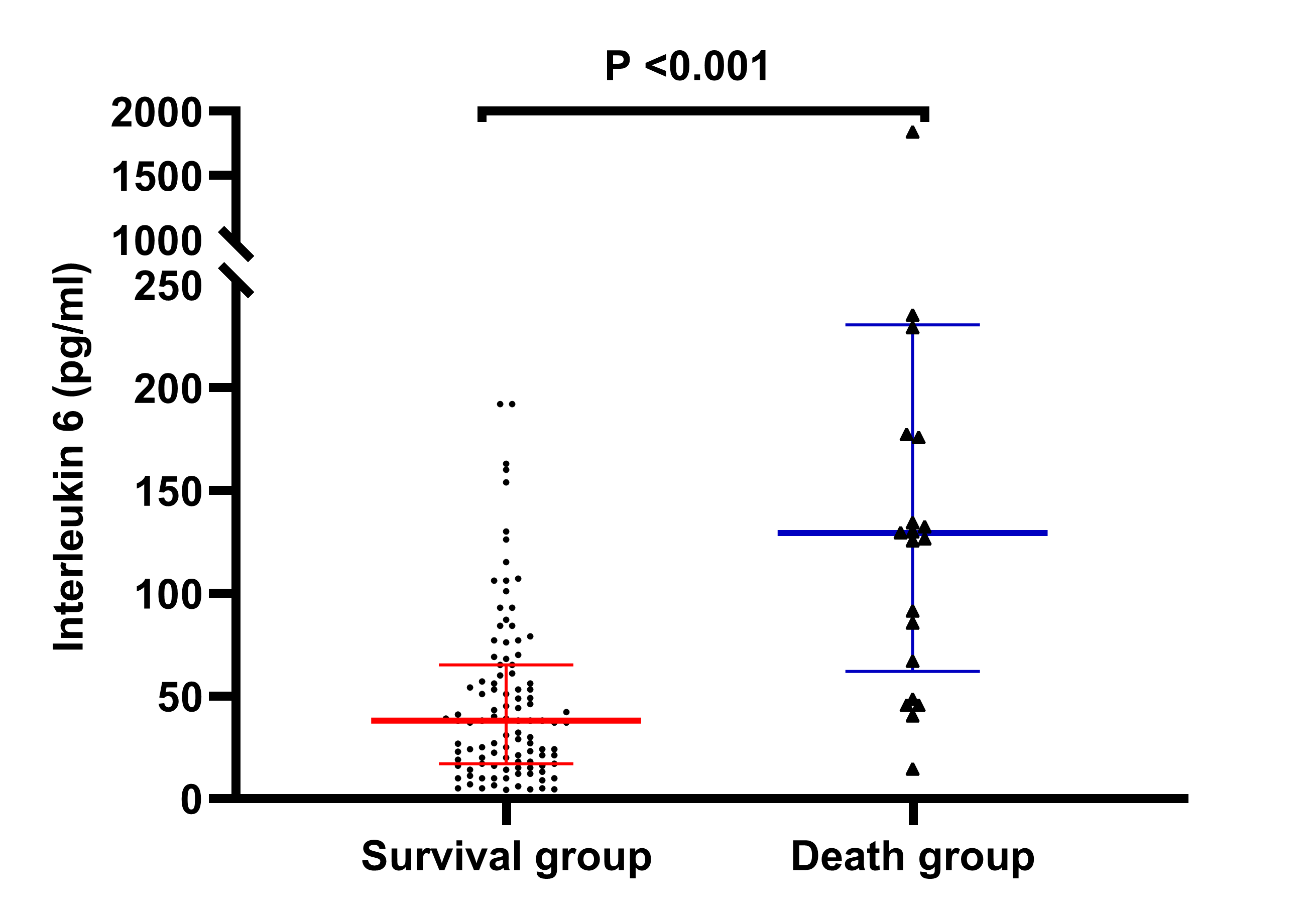

Supplement: S1 Fig — (TIF) [file pntd.0011158.s005.tif]
