# Supplementary material for: A new model for predicting the outcome and effectiveness of drug therapy in patients with severe fever with thrombocytopenia syndrome: A multicenter Chinese study
Source: PLoS Negl Trop Dis. 2023 Mar 6;17(3):e0011158. doi: 10.1371/journal.pntd.0011158 (PMC10019728; doi:10.1371/journal.pntd.0011158)
Supplement: Attachment — Submitted filename: 20221028-Reply.docx [file pntd.0011158.s007.docx]

*2022.10.28*

*PLoS Neglected Tropical Diseases*

Dear Editor:

We wish to re-submit our revised manuscript titled, "A new model for predicting the outcome and effectiveness of drug therapy in patients with severe fever with thrombocytopenia syndrome: A multicenter Chinese study." The manuscript ID is PNTD-D-22-00383- [EMID:818e15115cf12745].

We thank you and the reviewers for your thoughtful suggestions and insights. The manuscript has benefited from these helpful comments, and we look forward to working with you and the reviewers to move this manuscript closer to publication.

The manuscript has been rechecked and the necessary changes have been made in accordance with the reviewers’ suggestions. We hope these meet the approval of the reviewers. These corrections do not influence the content and framework of the paper. The responses to all comments have been prepared and given below. We have not listed all the revisions here, but have marked them in track changes mode in the revised manuscript. We have uploaded figure files to the Preflight Analysis and Conversion Engine (PACE) digital diagnostic tool

Thank you for your consideration. I look forward to hearing from you.

Sincerely,

Zhenhua Zhang PhD, Prof.

Department of Infectious Diseases

the Second Hospital of Anhui Medical University

Furong Road 678, 230601 Hefei, China

Tel/Fax number: 0086 551 65997113.

Email address: zzh1974cn@163.com

Reviewer #2: -Are the objectives of the study clearly articulated with a clear testable hypothesis stated? :YES

-Is the study design appropriate to address the stated objectives? : YES

-Is the population clearly described and appropriate for the hypothesis being tested? : YES

-Is the sample size sufficient to ensure adequate power to address the hypothesis being tested? : YES

-Were correct statistical analysis used to support conclusions? : YES

-Are there concerns about ethical or regulatory requirements being met? : YES

Results

-Does the analysis presented match the analysis plan? : YES

-Are the results clearly and completely presented? : YES

-Are the figures (Tables, Images) of sufficient quality for clarity? : YES

Conclusions

-Are the conclusions supported by the data presented? : YES

-Are the limitations of analysis clearly described?: YES

-Do the authors discuss how these data can be helpful to advance our understanding of the topic under study?: YES

-Is public health relevance addressed?: YES

Editorial and Data Presentation Modifications?:No Response

Reply: Thank you very much for your itemized evaluation.

Reviewer #2: - A report reported IL-6 and IL-10 levels, rather than viral load and neutralizing antibody titers, in patients with an SFTSV infection strongly correlated with outcomes (for severe disease with an ultimate outcome of recovery or death). Do authors also check cytokine in this study?

Reply: Thank you for your comment. We note that Jeong Rae Yoo found that IL-6 and IL-10 levels, rather than viral load and neutralizing antibody titers, were strongly correlated with outcomes (for severe disease with an ultimate outcome of recovery or death) in patients with an SFTSV infection. However, our study was a retrospective study that did not include these cytokines. We expect to include these cytokines and lymphocyte typing in future studies to better explore the mechanism of disease occurrence and to predict prognosis (Page 29, lines 495-498).

- Authors described “Treatments for patients with SFTS include ribavirin, gamma globulin administration, steroids, plasma exchange, and symptomatic supportive treatment on SFTS infection. In vitro studies have confirmed the anti-SFTSV effect of ribavirin (3, 9, 10). However, clinical studies have demonstrated that ribavirin does not significantly reduce mortality” in introduction. But, authors also described below“Ribavirin administration did not have a significant effect on mortality. However, a subgroup analysis revealed a significant treatment effect in the single-positive group (P = 0.006), but not in the double-positive and double-negative groups (both P > 0.99)”Could authors more detail describe the effect of Ribavirin in manuscript?

Reply: We apologize for not describing the role of ribavirin in each subgroup in detail in the manuscript, which led to your misunderstanding. As per your suggestion, the role of ribavirin has been described in more detail in the revised manuscript. (Page21, lines321-324).

- Authors described that the mortality rates of patients in the positive and negative subgroups for score of the joint indices were 36.2% and 1.1%, respectively and neurologic symptoms, age, SFTSV RNA, and gastrointestinal bleeding were independent risk factors affecting the prognosis of patients with SFTS. Are joint indices not independent risk factors affecting the prognosis of patients with SFTS?

Reply: We apologize for not describing this clearly. We have specified that neurological symptoms and joint indices score (including age, SFTSV RNA, and gastrointestinal bleeding) are independent risk factors for prognosis. (Page 17, lines 245-249)

Reviewer #1: In summary, the manuscript was designed to address an important question, however, the study provided no novel findings on improving clinical practice or refine the clinical diagnosis. the methodology is unclear, the writing could be significantly improved in terms of English language as well as overall cohesiveness and clarity. Finally, there are substantial data presented in the results that are not justified in the introduction nor described or defined in the methods. The manuscript is very sloppy constructed and needs to be edited to correct many typos and errors as well as many grammatical errors. Many sentences are incomplete and very difficult to understand.

Reply: Thank you for your guidance and suggestions. This retrospective multicenter study analyzed the data of 377 SFTS patients separated into a modeling group and a validation group, and we developed a simple model to predict mortality in SFTS patients. In particular, this model is of great significance in evaluating drug efficacy in SFTS patients.

The advantages of this study are as follows: It was a multicenter study which included a modeling group and a validation group. Neurological symptoms or signs are very important in predicting the prognosis of patients, but combined parameters (including age, viral load, and gastrointestinal bleeding) could be used as a valuable supplement. Our model demonstrated great efficiency in evaluating drug efficacy, clearly showing that the early initiation of ribavirin and/or prophylactic use of antibiotics will help improve prognosis in single-positive patients (i.e., those who were positive for one of the two indicators).

The manuscript has been revised according to your comments. The Introduction and Methods have been improved, and the English language and syntax errors have been submitted to a professional language modification company (Editage) for polishing. (Page 5, lines 72-83; Page 6, lines 92-98; Page 7, lines 109-125; Page 10, lines 158-170; Page11, lines 178-184.)

Abstract

Levels differed significantly between the infection group and the non-infection group: what do you mean by infection and non-infection group？Need to be clarified in the abstract.

Reply: The most common sites of infection observed in this study were the lungs and [bloodstream](javascript:;). The infected group included SFTS patients with pneumonia or sepsis, while the noninfected group included patients with no signs of infection. (Page 2, lines37-38)

Introduction

* There is only weak description of the background on the modelling study for the risk assessment of SFTS that cannot well support the hypothesis of the current research.

Reply: The description of the background on the modelling study for the risk assessment of SFTS has been expanded to support the hypothesis of the research. ((Page 5, lines 72-83; Page 6, lines 92-98)

* It does not make sense to claim “, these were all single-center studies with relatively small sample sizes….”, since the Ref 2, over 2000 patients were used for the modelling, despite of single center study, the model was of highly credibility based on large sample size.

Reply: I am deeply sorry for the inappropriate wording of this paragraph, which has been modified in the revised draft (Page 6, lines 98). Li et al.'s research is very valuable, and the sample size is large. The clinical model established also includes many parameters, which suggests that ribavirin antiviral therapy has clinical value.

The importance of viral load assessment has been reported in several studies, including Li et al.'s study, and our study had similar results. However, the clinical scoring system by Li et al. did not include the viral load in the model, which may be due to the fact that only some patients underwent quantitative virus detection(Page 6, lines 95-98).

More importantly, Li et al.'s study only evaluated the efficacy of ribavirin based on viral load stratification and did not evaluate the impact of gamma globulin and antibiotics on disease outcome. Our study stratified the severity of SFTS patients according to the model, and the results showed that ribavirin and antibiotics reduced the mortality of patients with SFTS, while gamma globulin may also have a therapeutic effect.

--"Physical examination showed tenderness in the upper abdomen and subxiphoid process, blood and urine amylase and lipase levels were significantly increased,” do you mean increased as compared with which group?

Reply: We apologize for not describing this clearly and have made revisions accordingly. The patients with SFTS in the pancreatitis group had epigastric and subxiphoid tenderness, and their hematuria amylase and lipase levels were significantly elevated, whereas patients without concurrent pancreatitis did not have these signs. We have also added a reference to the diagnostic criteria of pancreatitis in the revised manuscript. (Page11, lines 181-182)

Methods

-- In terms of methodology, it is unclear how the cases were approached and recruited and tested. How do you choose the diagnosis method among RT-PCR, IgM?

--The description of the case patient should be strengthened. Was the first blood sample of the case patient tested for SFTSV by IgM/IgG?

Reply: SFTS was diagnosed based on the criteria of the Guideline for Prevention and Treatment of Severe Fever with Thrombocytopenia Syndrome Version (2010). The diagnostic criteria for laboratory-confirmed SFTSV infection were acute fever with thrombocytopenia and detection of viral RNA and/or virus-specific IgM antibodies in the peripheral blood. The exclusion criteria were laboratory-confirmed infection with other pathogens, rickets disease, a history of acute or chronic blood disorders, malignant tumors, and AIDS and other immunodeficiency diseases (Page 9, lines 146-152).

To collect clinical data, we designed a medical record table containing information regarding demographic factors, epidemiology, clinical manifestations, physical examination, laboratory parameters, and date of onset. The data were entered by trained researchers. The patients who were discharged were followed up for 1 month after discharge (Page 9-10, lines 153-157).

SFTSV nucleic acid was detected by real-time quantitative RT-PCR using an SFTSV rRT-PCR kit purchased from Da An Gene Co., Ltd. The operation was performed in strict accordance with the kit and instrument operation procedures, and the detection limit was 1000 copies/mL. The forward primer of the real-time RT-PCR was 5'-TAAACTTCTGTCTTGCTGGCTCC-3', the reverse primer was 5'-TGGCAAGATGCCTTCACCA-3', and the probe was 5'-CGCATCTTCACATTGAT-3'. SFTSV-specific IgM and IgG antibodies were detected by the gold-standard method, and the kit was purchased from Wuxi Xinlianxin Biomedical Technology Co., Ltd. The first blood sample from each SFTS patient was tested for SFTSV RNA and IgM/IgG, and the results were used in this study. If the first blood sample tested negative for IgM, it was reviewed after the patient had recovered (Page10, lines 165-168). The description of the case patient have been strengthened (Page13, lines 201-207). We have modified the corresponding parts of our manuscript.

-- Please clarify which gene was used to determine the viral load and how?

Reply: SFTSV nucleic acid was detected by real-time quantitative RT-PCR. The primer and probe of the real-time RT-PCR test kit were taken from the S fragment of SFTSV. We compared the sequences of all genotypes in the literature and found that the amplified sequence is relatively conservative among all genotypes with little variation. The detection limit was 1000 copies/mL, and 1000 copies/mL were recorded if the test result was negative (Page10, lines 158-165).

--There is a severe concern that the high discrepancy among multiple medical centers on the treatment regimens, actually, it is suspicious that dugs listed in the paper might be of highly diversified for the administered dose or duration across the study hospitals

Reply: The three medical centers in this study were all affiliated hospitals of Anhui Medical University, with leading diagnosis abilities and treatment levels in the province. The treatment was carried out according to the Guideline for Prevention and Treatment of Severe Fever with Thrombocytopenia Syndrome Version (2010), and the drug doses were calculated and adjusted according to the body weight, liver function, and kidney function of the patients.

The laboratory parameters comprised complete blood count, biochemistry, and coagulation system tests. In regard to quality control management, the clinical laboratory divisions of these institutions periodically performed external quality control checks for each other. Therefore, the quality control of laboratory data was maintained among these institutions, and the three medical centers underwent quality control inspections by Anhui Clinical Laboratory Quality Control Center every quarter to ensure the data stability.

The three medical centers were analyzed separately, and there was no significant difference between the data from the three centers. (Page13, lines 208-212)

Results

--The demographic information of the patients are missing. Are the patients recruited from different hospitals comparable for the clinical data and the treatment?

Reply: Demographic data have been added to the results. The patients recruited in this study were mainly from the Anhui province, and had similar epidemiology. The clinical characteristics and test indicators of the modeling and validation groups were analyzed in Table S1; most of the data were consistent.

The review was conducted by two investigators who checked all the information against medical records. The data were entered by trained researchers, and all data were collected after treatment.

Quality control of laboratory data was maintained among the three medical institutions and inspected quarterly to ensure data stability.

Nucleic acid and antibody tests for patients with SFTS in the three hospitals were carried out by the same testing company.

-- Measurement of viral loads was described in a vague way, and how it was used in the score estimation was not described, was the copy/ml log transformed or otherwise?

Reply: Viral load measurements were recorded as copies/mL, and log10 transformed was used for conversion during statistics (Page 10, lines 162-164).

---Was the age used as a continuous variable? Was the Gastrointestinal bleeding used as Yes (1 score ) and no (0 Score)? If yest 30X0 is still 0

Reply: Age was used as a continuous variable. According to the logistic regression rules, gastrointestinal bleeding was classified as a categorical variable. Gastrointestinal bleeding was recorded as yes (30 scores) and no (0 score), indicating that the weight of bleeding in the model is very large(Page17, lines 249).

---It seems rather arbitrary to exclude neurologic symptoms as a variable because it has a huge OR, then reused it as a separate variable in the positive counting. It seems the most contribution of the death was due to the presence of neurological, considering the mortality rate of 79.3% versus 6.8% between double positive and single-positive. Then why all the efforts to establish another multiple variable score?

Reply: We also noticed that neurologic symptoms as a variable have a huge OR by statistical analysis because they have an overwhelming influence. If we directly conduct a multivariate analysis with other variables, the other variables show no statistical significance. However, many previous studies have found that age, viral load, bleeding, and other factors also have good predictive value for the prognosis of the disease. Therefore, upon discussion with the statistical expert Professor Fen Huang (Department of Statistics, Anhui Medical University, Meishan Road 81, Hefei 230032, China. Phone:13956950811; Email: fenh@ahmu.edu.cn), we believe that the combined application of other factors may enhance their weight in the prediction. Therefore, after excluding symptoms of the nervous system, we established a logistic regression model through multifactor analysis. The results showed that, compared with the three variables alone, the neurological symptoms had absolute predictive values. However, the joint index scores of the three variables combined increased the predictive values, and the consistency between the neurological symptoms and the joint index scores was statistically different. Therefore, the combined index and the neurological symptoms had their own advantages and complementary effects in predicting the prognosis of patients.

In addition, neurological symptoms often occur late in the disease, and joint index scores including age, SFTSV RNA, and gastrointestinal bleeding can be identified or detected and evaluated early.
