# Supplementary material for: A new model for predicting the outcome and effectiveness of drug therapy in patients with severe fever with thrombocytopenia syndrome: A multicenter Chinese study
Source: PLoS Negl Trop Dis. 2023 Mar 6;17(3):e0011158. doi: 10.1371/journal.pntd.0011158 (PMC10019728; doi:10.1371/journal.pntd.0011158)
Supplement: Attachment — Submitted filename: renamed_9181c.docx [file pntd.0011158.s008.docx]

*2023.02.03*

Shaden Kamhawi, Paul Brindley, & Peter J. Hotez

Editors-in-Chief

*PLoS Neglected Tropical Diseases*

Dear Editors:

Due to the Chinese Spring Festival and we apologize for the delay in revising the manuscript titled, "**A new model for predicting the outcome and effectiveness of drug therapy in patients with severe fever with thrombocytopenia syndrome: A multicenter Chinese study**." The manuscript ID is PNTD-D-22-00383- [EMID:818e15115cf12745].

We thank you and the reviewers for your thoughtful suggestions and insights. The manuscript has benefited from these helpful comments, and we look forward to working with you and the reviewers to move this manuscript closer to publication.

The manuscript has been rechecked, and the necessary changes have been made in accordance with the reviewers’ suggestions. In addition, we are very sorry to find that Shanshan Sun's authorship unit is incorrectly marked, which has been revised in this edition of the manuscript. We hope these meet the approval of the reviewers. These corrections have not influenced the content and framework of the paper. The responses to all comments have been prepared and given below. We have not listed all the revisions here, but have marked them in track changes mode in the revised manuscript. Thank you for your consideration. I look forward to hearing from you.

Sincerely,

Zhenhua Zhang PhD, Prof.

Department of Infectious Diseases

the Second Hospital of Anhui Medical University

Furong Road 678, 230601 Hefei, China

Tel/Fax number: 0086 551 65997113.

Email address: zzh1974cn@163.com

1) We have noticed that you have included SI Legends but "Fig S1 IL-6.tif" is missing in the file. Please add a full list of legends for your Supporting Information file after the references list.

**Response**: Thank you for your suggestion. A full list of legends for "S1 Fig IL-6.tif" has been added after the reference list.

2) Please amend your detailed Financial Disclosure statement. This is published with the article, therefore should be completed in full sentences and contain the exact wording you wish to be published.

i). State the initials, alongside each funding source, of each author to receive each grant.

**Response:** We are very sorry that due to our misunderstanding, the financial disclosure section on the webpage was incorrectly filled in. However, the research was conducted in the absence of any commercial or financial relationships that could be construed as a potential conflict of in interest and this has been modified on the webpage.

3) Please ensure that the funders and grant numbers match between the Financial Disclosure field and the Funding Information tab in your submission form. Note that the funders must be provided in the same order in both places as well. Currently, your Funding Information is missing few of the grants and authors listed in your Financial Disclosure.

**Response:** As previously noted, this study did not receive any commercial or financial relationships that could be construed as a potential conflict of in interest. This has been accordingly modified on the webpage.

4) We have noticed that your Financial Disclosure is duplicated in the submission.

Financial Disclosure 1: " The study was supported by Anhui Provincial Natural Science Foundation (2108085MH298), The Second Hospital of Anhui Medical University Natural Science Foundation (2019GMFY02), Clinical Medicine project of Anhui Medical University (2021lCXK027), and the National Science Foundation of China (81273142). "

Financial Disclosure 2: " The study was supported by the Anhui Provincial Natural Science Foundation (2108085MH298), The Second Hospital of the Anhui Medical University Natural Science Foundation (2019GMFY02), the Clinical Medicine Project of Anhui Medical University (2021lCXK027), and the National Science Foundation of China (81273142). The funders had no role in the study design, data collection and analysis, decision to publish, or preparation of the manuscript "

Could you please confirm which of these two statements is appropriate for this submission?

**Response**: We are sorry about that. One of the duplicate financial disclosures has been removed from the submission and the retained one resubmitted.

5) In the online submission form you indicate that your data is not available for proprietary reasons and have provided a contact point for accessing this data. Please note that your current contact point is a co-author on this manuscript. According to our Data Policy, the contact point must not be an author on the manuscript and must be a third party. Please revise your data statement to a non-author institutional point of contact, such as a data access or ethics committee, and send this to us via return email. Please also include contact information for the third party organization, and please include the full citation of where the data can be found.

**Response:** We apologize for any confusion. All relevant data openly available in a public repository. This has been accordingly modified on the webpage. I'm sorry to bother you and there exist a question before I approve in the last step of the submission process. When the PDF file was generated, an error occurred. In the section of “Data Availability”, I changed the “Data cannot be shared publicly due to the enrollment agreement with the included patients. Data are available from the correspond author (Second hospital of Anhui Medical University) for researchers who is interest in our data.” to “All relevant data openly available in a public repository”. However, the original content is still retained in the retained PDF version and there was exist an extra paragraph behind “Data Availability” with title of “Describe where the data may be found in full sentences. If you are copying our sample text, replace any instances of XXX with the appropriate details” which should not exist (Page 6). I have no idea for removing the paragraph and modifying this error. Could you please help me solve this problem? I looking forward to your reply.
